# Current Advances in N6-Methyladenosine Methylation Modification During Bladder Cancer

**DOI:** 10.3389/fgene.2021.825109

**Published:** 2022-01-11

**Authors:** Qiang Liu

**Affiliations:** Department of Urology, Cancer Hospital of China Medical University, Liaoning Cancer Hospital and Institute, Shenyang, China

**Keywords:** N6-methyladenosine, “writers”, “erasers”, “readers”, methylation, bladder cancer

## Abstract

N6-methyladenosine (m6A) is a dynamic, reversible post-transcriptional modification, and the most common internal modification of eukaryotic messenger RNA (mRNA). Considerable evidence now shows that m6A alters gene expression, thereby regulating cell self-renewal, differentiation, invasion, and apoptotic processes. M6A methylation disorders are directly related to abnormal RNA metabolism, which may lead to tumor formation. M6A methyltransferase is the dominant catalyst during m6A modification; it removes m6A demethylase, promotes recognition by m6A binding proteins, and regulates mRNA metabolic processes. Bladder cancer (BC) is a urinary system malignant tumor, with complex etiology and high incidence rates. A well-differentiated or moderately differentiated pathological type at initial diagnosis accounts for most patients with BC. For differentiated superficial bladder urothelial carcinoma, the prognosis is normally good after surgery. However, due to poor epithelial cell differentiation, BC urothelial cell proliferation and infiltration may lead to invasive or metastatic BC, which lowers the 5-years survival rate and significantly affects clinical treatments in elderly patients. Here, we review the latest progress in m6A RNA methylation research and investigate its regulation on BC occurrence and development.

## Introduction

Bladder cancer (BC) is one of the most common malignant tumors of the urinary system; it ranks first among urological tumors in terms of incidence rate, and is the 9th highest incidence cancer in the world (Lenis et al., 2020; Li et al., 2021a). In recent years, BC treatment strategies have improved such that surgical resection combined with radiotherapy or chemotherapy are highly effective treatments (Jain et al., 2021; Tran et al., 2021). However, while immunotherapy has demonstrated strong prospects for solid tumor treatment, it remains to be clinically applied to BC (Afonso et al., 2020; van Puffelen et al., 2020; Wu and Abraham 2021). Immunotherapy is limited as it inhibits non-muscular and muscular invasive BC at the laboratory level (Schneider et al., 2019; Witjes et al., 2021). For patients with non-muscle invasive tumors (NMIBC), transurethral resection combined with postoperative bladder perfusion chemotherapy or BCG treatment strategies is usually adopted (Charpentier et al., 2021; Li et al., 2021e). However, 20–30% of NMIBC patients will progress to muscle invasive bladder cancer (MIBC), and 50% will develop distant metastases within 2 years of radical surgery (Jiang et al., 2021a; Liu et al., 2021a). For locally advanced or advanced MIBC patients, gemcitabine combined with cisplatin (GC regimen) remains the standard treatment, however, BC fatality rates have only dropped by 1.5% in the past 15 years (Kaur et al., 2021; Roviello et al., 2021). Due to its high recurrence and metastasis rate, the 5-years survival rate for patients with MIBC remains very low (Meeks et al., 2020; Patel et al., 2020; Jiang et al., 2021a). Therefore, while novel treatment strategies must be explored and BC molecular mechanisms clarified, recent evidence has suggested that m6A mechanisms actively participate in BC (Mu et al., 2021).

N6-methyladenosine (m6A) is one of the most common internal transcription modifications in eukaryotic messenger RNA (mRNA) (Huang et al., 2021; Oerum et al., 2021). The molecule was first identified in the 1970s, but recent studies have shown that m6A-associated mutations are closely related to BC occurrence (Liu et al., 2021b). In 2011, the fat-mass and obesity-associated protein (FTO) was reported to have functions in m6A demethylase and suggested that m6A modification was dynamically reversible (Zheng et al., 2020; Gu et al., 2021a; Tan et al., 2021; Zhao et al., 2021). Studies have since summarized the related modifications of m6A as methyltransferase complexes, demethylases, and corresponding readers coordinated regulation, which are classified as “writers,” “erasers,” and “readers,” respectively (Tang et al., 2021). M6A is abundant in 3′ untranslated regions (UTRs), stop codons, and long exon regions. The process has a high degree of evolutionary conservation, but with unclear biological functions (Yao et al., 2021; Zhao et al., 2021). M6A is co-catalyzed by the methylation modification enzymes, METTL3 and METL14. Also, WTAP and KIAA1429 function as m6A regulators to participate in catalytic processes (Wu et al., 2020; He and He 2021). Interestingly, the METTL3-METTL14 complex is more potent than individual components in catalyzing m6A formation (Song et al., 2021a; Uddin et al., 2021). M6A methylation is also demethylated by the FTO and AlkB homolog 5 (ALKBH5) demethylases (Ye et al., 2021). M6A modification is involved in all mRNA metabolic processes, including maturation, transport, splicing, translation, and degradation (Song et al., 2020; Li et al., 2021b Li et al., 2021c; Lou et al., 2021). M6A RNA methylation exerts critical biological functions in mammals, such as tissue development, circadian rhythms, DNA damage responses, gender identification, and tumor occurrence and development (Xu et al., 2020a; Li et al., 2020; Ma and Ji 2020; Gu et al., 2021b; Wu and Wang 2021). In this review, we discuss the potential mechanisms of m6A methylation-related regulators in BC initiation and development.

## M6A Methylation Regulators

**FIGURE 1:**
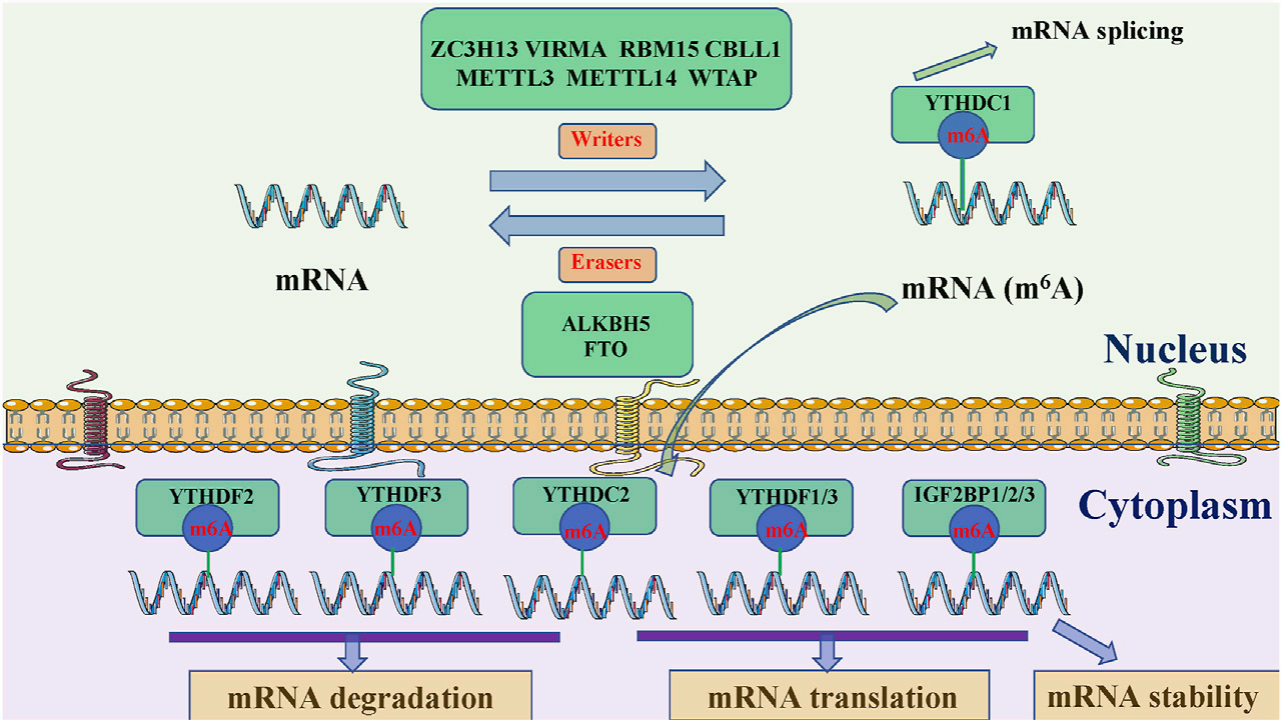
Potential m6A methylation mechanisms in RNA. M6A methylation is catalyzed by the writer complex, including METTL3, METTL14, WTAP, VIRMA, RBM15, ZC3H13, and CBLL1. The demethylases, FTO and ALKBH5 remove m6A modifications. Reader proteins (YTHDC1, YTHDF2, YTHDF3, YTHDC2, YTHDF1/3, and IGF2BP1/2/3) recognize m6A and determine target RNA targets. METTL3, methyltransferase 3, N6-adenosine-methyltransferase complex catalytic subunit; METTL14, methyltransferase 14, N6-adenosine-methyltransferase subunit; WTAP, WT1 associated protein; VIRMA, vir like m6A methyltransferase associated; RBM15, RNA binding motif protein 15; ZC3H13, zinc finger CCCH-type containing 13; CBLL1, Cbl proto-oncogene like 1; FTO, FTO α-ketoglutarate dependent dioxygenase; ALKBH5, alkB homolog 5, RNA demethylase; YTHDC1/2, YTH domain containing 1/2; YTHDF1/2/3, YTH N6-methyladenosine RNA binding protein 1/2/3; IGF2BP1/2/3, insulin like growth factor 2 mRNA binding protein 1/2/3.

M6A modification adds a methyl group to the N6 position of adenosine and is an evolutionarily conserved RNA modification (Han and Choe 2020; Zhang et al., 2020; Zhou et al., 2020). Approximately 0.3% of adenosine in mRNA is modified by m6A, with an average of three m6A modification sites in every transcript. M6A methylation mainly occurs in RRACH sequences (where R = A or G, H = A, C, or U), stop codons, 3′UTRs, and internal long exons, to regulate RNA transcription, processing, translation, and metabolism (Huang et al., 2020a; Chen and Wong 2020; Liang et al., 2020; Scarrow et al., 2020). The modification is controlled by m6A regulatory enzymes, amongst which, methyltransferases or m6A “writers” actively catalyze modifications, m6A “erasers,” with demethylase activity, eliminate m6A modifications, and m6A “readers” recognize modification (He et al., 2019; Huang et al., 2020b; Lee et al., 2020; Zhao et al., 2020) bases and convey information, thereby establishing an efficient and orderly m6A regulatory network (Figure 1).

The methyltransferase complex primarily includes methyltransferase-like 3 (METTL3), METTL14, vir like m6A methyltransferase associated (VIRMA), RNA binding motif protein 15 (RBM15), zinc finger CCCH-type containing 13 (ZC3H13), Cbl proto-oncogenes like 1 (CBLL1), and Wilm’s tumor 1-associated protein (WTAP). All proteins co-ordinate and regulate m6A control (Chen et al., 2019a; Ma et al., 2019; Williams et al., 2019). METTL3 functions as a core component where METTL14 combines with it to form a stable heterodimer to catalyze m6A RNA methylation *via* synergistic effects (Chen et al., 2019b; Yue et al., 2019). WTAP anchors the METTL3/14 complex on target RNA and promotes its nuclear accumulation (Lan et al., 2019; Liu et al., 2019). The KIAA1429-RBM15 complex was recently verified as a new component of the m6A “writer” complex, while RBM15 recruits the complex to target sites (Niu et al., 2018; Wang et al., 2018). METTL16 is also a novel m6A molecule targeting U6 small nuclear RNA (snRNA) and regulates S-adenosylmethionine homeostasis by elevating S-adenosylmethionine synthase expression during methionine starvation (Frye et al., 2018; Yang et al., 2018; Zhang 2018).

M6A demethylases include FTO and ALKBH5. FTO was identified as regulating steady-state energy levels and positively correlating with obesity risk (Deng et al., 2018a; Huang and Yin 2018). ALKBH5 is a homolog of FTO, and belongs to the Fe^2+^and α-ketoglutarate-dependent AlkB oxygenase family (Deng et al., 2018b; Dai et al., 2018). FTO and ALKBH5 both recognize m6A-modified nuclear RNA as a substrate, and catalyze the removal of m6A methyl modifications (Meyer and Jaffrey 2017; Wang et al., 2017).

M6A reading proteins are divided into three categories: proteins contain an evolutionarily conserved YTH domain which folds into a hydrophobic aromatic structure directly binding to m6A (Liao et al., 2018; Patil et al., 2018). YTH domain proteins are composed of YTHDF (YTHDF1, YTHDF2, and YTHDF3) and YTHDC subtypes (YTHDC1 and YTHDC2). YTHDF subtype proteins are mainly distributed in the cytoplasm.

Heterogeneous nuclear ribonucleoproteins (hnRNPs) mainly include three types, namely hnRNPC, hnRNPG, and hnRNPA2B1. The “m6A switch” phenomenon disrupts RNA hairpin structures and exposes single-stranded hnRNP binding motifs (Aguilo and Walsh 2017; Wu et al., 2017). These proteins bind to transcripts containing m6A via the m6A switch, thereby affecting mRNA localization and alternative splicing (Batista 2017; Roignant and Soller 2017).

Insulin-like growth factor 2 mRNA-binding proteins 1–3 (IGF2BP1–3) also recognize the GGC (m6A) sequences via the K homology domain, and enhance the stability and translation of downstream mRNAs in an m6A-dependent manner under normal and stress conditions (Adhikari et al., 2016).

## M6A Roles and Disease Mechanisms

**FIGURE 2:**
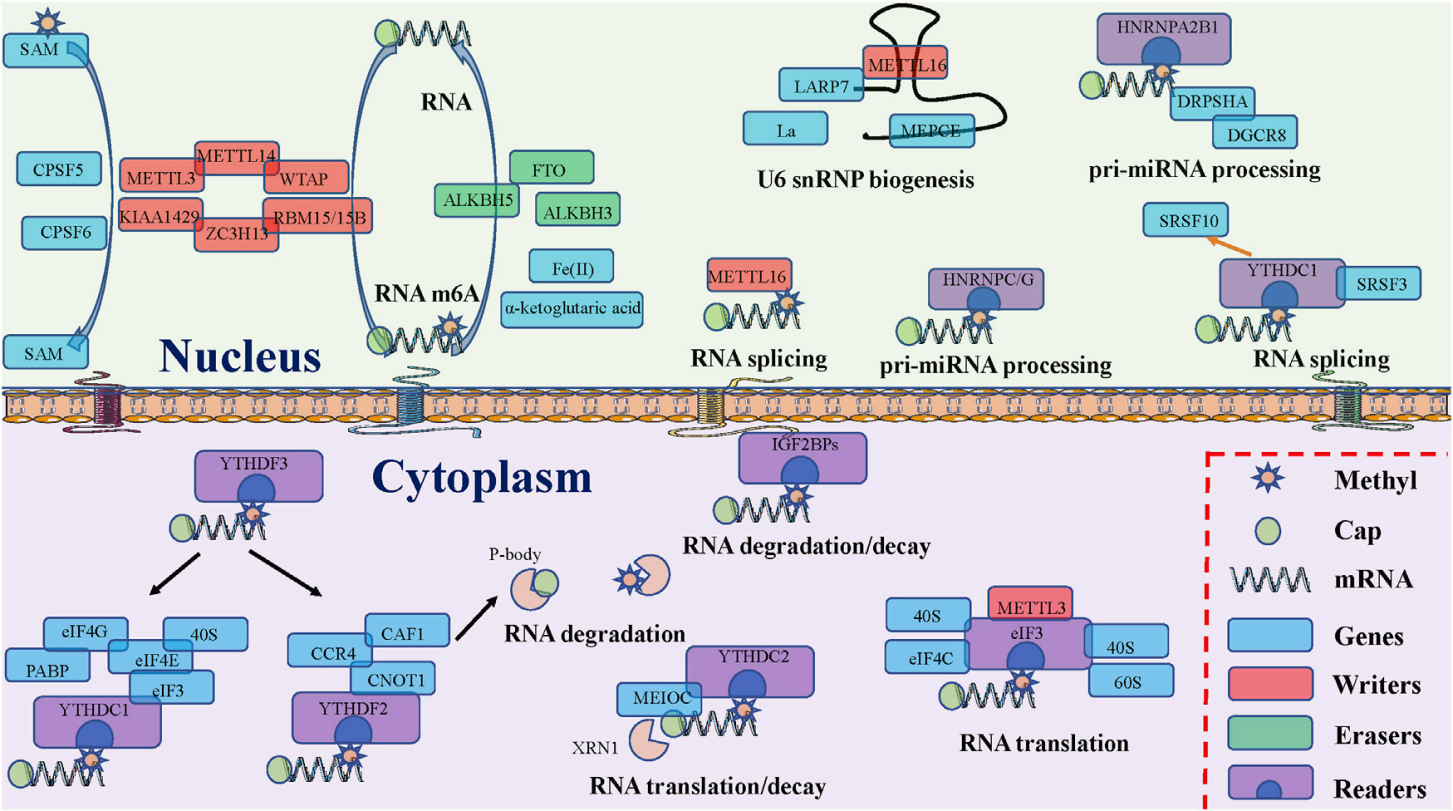
The potential functions of RNA m6A modification related proteins. “Writers,” “Erasers”, and “Readers” rely on several crucial factors to install, remove, and recognize m6A modifications and participate in various RNA metabolism steps, including splicing, export, translation, degradation, and decay.

The potential roles and mechanisms of m6A-related regulators are shown (Figure 2).

### M6A Methyltransferases

RNA m6A methylation is controlled by METTL3, METTL14, WTAP, RBM15 RBM15B, and Cbl proto-oncogene E3, and is catalyzed by methyltransferase complexes composed of CBLL1, VIRMA, and ZC3H13. Of these proteins, METTL3 and METTL14 exhibit m6A methyltransferase activity (Zhou et al., 2021a; Maldonado Lopez and Capell 2021; Pan et al., 2021). WTAP promotes m6A functionby recruiting METTL3 and METTL14 into nuclear speckles (Deng et al., 2021). RBM15 and RBM15B bind METTL3 and WTAP and guides them to specific RNA sites for m6A modification (Meng et al., 2021). VIRMA preferentially mediates mRNA methylation near 3′UTR and stop codon regions (Zhu et al., 2021a). ZC3H13, together with other cofactors such as WTAP, control nuclear m6A methylation (Zhou et al., 2021b).

METTL16 is a novel RNA methyltransferase that independently induces the m6A modification of the 3′UTR of mRNAs (Satterwhite and Mansfield 2021), which have crucial roles in maintaining mRNA stability and splicing. M6A methyltransferases also display carcinogenic roles in several cancers. METTL3-induced miR-222-3p up-regulation suppresses STK4 and promotes malignant behaviors in thyroid carcinoma cells (Lin et al., 2021). METTL3 also up-regulates the m6A modification of adenomatous polyposis coli (APC), leading to its mRNA degradation. Decreased APC increases β-catenin, cyclin D1, c-Myc, and PKM2 expression, resulting in mouse aerobic glycolysis, cell proliferation, and enhanced esophageal squamous cell carcinoma (ESCC) formation (Wang et al., 2021a). METTL3 also induces PLX4032 resistance to melanoma by promoting m6A-dependent EGFR translation (Bhattarai et al., 2021). METTL14 also aggravates podocyte injury and glomerulopathy progression *via* N-methyladenosine-dependent Sirt1 down-regulation (Lu et al., 2021). METTL14 promotes glomerular endothelial cell injury and diabetic nephropathy *via* m6A modification of the α-klotho protein (Li et al., 2021d). METTL16 promotes cell proliferation by up-regulating cyclin D1 expression in gastric cancer (Wang et al., 2021b). WTAP up-regulation reduces PERP levels *via* m6A modification, which in turn promotes pancreatic cancer growth and metastasis (Wang et al., 2020a). WTAP expression is significantly increased in HCC and promotes liver cancer development. WTAP-guided m6A modifications may also promote HCC progression *via* the HuR-ETS1-p21/p27 regulatory axis (Chen et al., 2019c).

### M6A Demethyltransferase

RNA m6A methylation is a reversible process, with its demethylation reliant on demethylases. FTO catalyzes m6A demethylation and displays strict substrate selectivity near alternatively spliced exons and poly-A sites (Lan et al., 2020; Jiang et al., 2021b; He and He 2021). ALKBH5 functions with FTO to ensure balanced m6A modifications in the transcriptome (Chen et al., 2021a; Purslow et al., 2021; Wu et al., 2021). ALKBH3 was identified as another m6A demethylase with easier binding to tRNA m6A sites than mRNA or rRNA sites (Esteve-Puig et al., 2021; Wollen et al., 2021). FTO expression is increased in breast cancer and promotes cell growth and metastasis (Niu et al., 2019). FTO also mediates m6A demethylation in the 3′UTR of BNIP3 mRNA and induces its degradation *via* a YTHDF2 independent manner. The FTO-mediated epigenetic up-regulation of LINC00022 also promotes tumorigenesis in ESCCs (Cui et al., 2021). ALKBH5-HOXA10 loop-mediated JAK2 m6A demethylation causes cisplatin resistance in epithelial ovarian cancer (Nie et al., 2021). ALKBH5 promotes the cadmium-induced transformation of human bronchial epithelial cells by regulating PTEN expression in an m6A-dependent manner (Li et al., 2021e). ALKBH5 was also identified in cell and animal models as related to patient prognoses and the suppression of esophageal cancer malignancies. The protein also demethylates pri-miR-194-2 and inhibits it in an m6A/DGCR8-dependent manner (Chen et al., 2021b).

### M6A Binding Proteins

M6A modifications exert biological functions by binding to m6A-binding proteins (Dai et al., 2021; Tsuchiya et al., 2021). YTHDF1 knockout reduces the overall level of IFN-induced A-to-I RNA editing, thereby activating the Double stranded RNA sensing pathway and promoting IFN-stimulated gene expression (Terajima et al., 2021). YTHDF1 deficiency also inhibits viral replication in cells by modulating IFN responses. YTHDF2 inhibits cardiac hypertrophy through a Myh7 mRNA decoy in an m6A-dependent manner (Xu et al., 2021). YTHDF1 also correlates with the immune microenvironment and predicts clinical outcomes and therapeutic efficacy in breast cancer (Hu et al., 2021). YTHDF1 and YTHDF2 are associated with better patient survival rates and an inflamed tumor-immune microenvironment in non-small-cell lung cancer (Tsuchiya et al., 2021). Highly expressed YTHDF3 promotes cancer cell interactions with brain endothelial cells and astrocytes, blood-brain barrier extravasation, angiogenesis, and growth (Chang et al., 2020). Mechanistically, YTHDF3 enhances the translation of m6A-rich ST6GALNAC5, GJA1, and EGFR transcripts. MiR-30d is a new target modified by YTHDC1 via m6A, with miR-30d inhibiting pancreatic tumors by inhibiting aerobic glycolysis (Hou et al., 2021). YTHDC2 contains an RNA helicase domain, recognizes m6A methylated adenosine at nucleotide 331, and cooperates with the cellular La antigen to support HCV IRES-dependent translation (Kim and Siddiqui 2021).

## M6A Roles and Mechanisms in BC

Recent studies reported that m6A-modified mRNA is dysregulated in several cancers, with *in vivo* and *in vitro* anti-cancer effects identified. Dysregulated m6A-related factors may alter m6A modifications in tumors and interfere with cancer progression. In the following sections, we summarize m6A regulatory factor roles in BC (Table 1).

**TABLE 1:** The role of RNA m6A modification in bladder cancer.

Type	m6A regulator	Role in cancer	Biological function	Mechanism	References
m6A writer	METTL3	Oncogene	Promotes cell growth and invasion	METTL3/AFF4/NF-κB/MYC	Lan et al. (2020)
	METTL3	Oncogene	Promotes malignant transformation and tumorigenesis	METTL3-m6A-CDCP1	Purslow et al. (2021)
	METTL3	Oncogene	Promotes cell proliferation	METTL3-DGCR8-PTEN	Chen et al. (2021b)
				METTL3/pri-miR221/222	
	METTL3	Oncogene	Promotes cancer proliferation and metastasis	METTL3/YTHDF2/SETD7/KLF4	Wu et al. (2021)
	METTL3	Oncogene	Promotes bladder cancer development	METTL3-m6A-CDCP1	Wollen et al. (2021)
	METTL3	Oncogene	Promotes oncogenesis and tumor angiogenesis	METTL3/TEK/VEGF-A	Esteve-Puig et al. (2021)
	METTL3	Oncogene	Promotes tumor proliferation and metastasis	cisplatin/METTL3/G-CSF	Niu et al. (2019)
	METTL14	Tumor suppressor	Inhibits the proliferation, self-renewal, metastasis and tumor initiating capacity of bladder TICs	METTL14/m6A/NOTCH1	Cui et al. (2021)
	METTL14	Tumor suppressor	Inhibits cell invasion	ISO/FOXO3a/METTL14/Vimentin	Nie et al. (2021)
m6A eraser	FTO	Tumor suppressor	Inhibits cell proliferation and invasion	—	Li et al.(2021b)
	FTO	Oncogene	Promotes cancer initiation and progression	UPS18/FTO/PYCR1	Dai et al. (2021)
	FTO	Oncogene	Stimulates cell viability and tumorigenicity	FTO/MALAT/miR-384/MAL2	Tsuchiya et al. (2021)
	ALKBH5	Tumor suppressor	Inhibits bladder cancer growth and progression	ALKBH5/ITGA6/YTHDF1/3	Terajima et al. (2021)
	ALKBH5	Tumor suppressor	Inhibits cell proliferation, migration, invasion and increases cisplatin chemosensitivity	ALKBH5/m6A/CK2a	Xu et al. (2021)
m6A reader	YTHDF1/3	Oncogene	Promotes bladder cancer growth and progression	METTL3/ITGA6/YTHDF1/3	Hu et al. (2021)
	YTHDF2	Oncogene	Promotes cancer proliferation and metastasis	METTL3/YTHDF2/SETD7/KLF4	Chang et al. (2021)
	IGF2BP1	Oncogene	Promotes bladder cancer cell invasion, metastasis and cell cycle progression	circPTPRA/IGF2BP1/FSCN1- MYC	Hou et al. (2021)
	IGF2BP3	Oncogene	Promotes cell proliferation, cell cycle and inhibit apoptosis	IGF2BP3/JAK/STAT	Kim and Siddiqui (2021)

### M6A Modification of Related Protein Expression Up-Regulates METTL3 in BC

METTL3 was the first discovered methyltransferase and forms a complex with METTL14 and WTAP to promote RNA methylation. METTL3 in human tissue is highly expressed and conserved, especially in the testes. Recent studies reported that METTL3 is significantly highly expressed in chronic myeloid leukemia (Ianniello et al., 2021), thymic epithelial tumors (Iaiza et al., 2021), esophageal cancer (Han et al., 2021), and prostate cancer (Chen et al., 2021b), suggesting a close relationship with malignant tumor development. Previous studies also suggested that METTL3 is significantly up-regulated in BC. METTL3 knockdown significantly reduces BC proliferation, invasion, and survival rates *in vitro*, and tumorigenicity *in vivo*. In contrast, METTL3 overexpression promotes BC cell growth and invasion (Cheng et al., 2019). AF4/FMR2 are two critical regulators of the NF-κB pathway (IKBKB and RELA) and MYC and were verified as downstream targets of METTL3-mediated m6A modification. Yang et al. reported that METTL3 and CDCP1 were up-regulated in BC tissue, and their expression levels were interrelated with respect to BC progression (Yang et al., 2019). METTL3-m6A-CDCP1 axis repression inhibits the growth and progression of chemically transformed and BC cells. This axis and chemical carcinogens exert a synergistic impact on promoting the malignant transformation of urothelial cells and BC occurrence. Han et al. (2019) indicated that METTL3 exerts carcinogenic effects in BC by interacting with DGCR8 and positively regulating pri-miR221/222 processes in an m6A-dependent manner. Xie et al. (2020) discovered that the tumor-promoting functions and specific regulatory mechanisms of the m6A axis are composed of the core “writer” protein, METTL3 and the main “reading” protein, YTHDF2. METTL3 consumption damages cancer proliferation and metastasis. The METTL3/YTHDF2 m6A axis directly degrades the mRNA of the tumor suppressors, SETD7 and KLF4 and promotes BC development. Ying et al. (2020) showed that the RCas9-METTL3 system mediates the effective site-specific m6A installation on CDCP1 mRNA and promotes BC progression. Wang et al. (2021c) suggested that METTL3 absence inhibits tyrosine kinase endothelium (TEK) and vascular endothelial growth factor A (VEGF-A) by reducing the abundance of m6A peaks at specific sites. METTL3 consumption down-regulates mRNA and protein expression levels of TEK and VEGF-A. Also, activation of TEK-VEGF-A-mediated tumor development and angiogenesis requires METTL3-mediated m6A modification. Mu et al. reported that cisplatin blocks G-CSF methylation by targeting METTL3 and reducing fibrocystic-myeloid-derived suppressor cells during IAIC (Mu, et al., 2021).

#### 4.1.1 FTO

FTO is the first obesity susceptibility gene confirmed by whole genome scanning and is localized to human chromosome 16q 12.2, is approximately 430 kb, and contains nine exons and eight introns. The protein is widely expressed in the hypothalamus, adipose tissue, pancreatic islets, and other tissues (Zarza-Rebollo et al., 2021) (Zhou et al., 2021b). Song et al. (2021b) showed that USP18 post-translational deubiquitination up-regulates FTO protein expression, while FTO promotes BC occurrence and progression *via* its demethylase activity on PYCR1 to stabilize its transcript. Thus, the UPS18/FTO/PYCR1 signaling network could act as a potential therapeutic target for BC. In addition, FTO regulates the MALAT/miR-384/MAL2 axis via m6A RNA modification to initiate BC. Thus, FTO has the potential to be a prognostic biomarker for BC (Tao et al., 2021).

#### 4.1.2 IGF2BP1

Several IGF2BP molecules were identified thanks to molecular detection and proteomic approaches. These proteins exert key biological roles in cell polarization, proliferation, migration, and differentiation, and are closely related to the development of many tumors (Bell et al., 2013). The IGF2BP family includes IGF2BP1, IGF2BP2, and IGF2BP3, and all of which are highly conserved onco-embryonic proteins mainly expressed in embryonic tissue. Their expression levels are extremely low, or negligible in adult tissue (Du et al., 2021). Xie et al. (2021) showed that IGF2BP1 binds circPTPRA in the BC cell cytoplasm, with the ectopic expression of circPTPRA eliminating the promotion of IGF2BP1-induced growth and metastasis in BC cells.

#### 4.1.3 IGF2BP3

Huang et al. (2020c) reported that IGF2BP3 expression is elevated in BC tissue and is closely related to a poor prognosis in BC patients. Overexpressed IGF2BP3 significantly promotes cell cycle and BC cell proliferation by activating the JAK/STAT signaling pathway and inhibiting apoptosis.

### 4.2 M6A Modification of Related Protein Expression Down-Regulates METTL14 in BC

Gu at al. reported that METL14 expression decreases in BC and bladder tumor-initiating cells (TIC). METL14 knockout significantly promotes cell proliferation, self-renewal, metastasis, and tumor initiation of bladder TIC (Gu et al., 2019). METTL14 and m6A modifications are involved in Notch1 mRNA stability. In addition, isorhapontigenin reduces vimentin protein levels by increasing METTL14 expression and up-regulating METTL14 mRNA by activating the transcription factor, FOXO3a, thereby impacting on BC progression (Zhang et al., 2021).

#### 4.2.1 FTO

Using real-time fluorescent quantitative PCR and TCGA analysis, Wen et al. (2020) observed that FTO mRNA expression levels in urothelial BC are significantly lower than normal tissue. FTO knockdown significantly promotes the proliferation and migration of 5,637 and T24 cells (Wen et al., 2020).

#### 4.2.2 ALKBH5

ALKBH5 is an RNA demethylation modification enzyme during m6A modification processes (Cai et al., 2021; Peng et al., 2021; Wu, et al., 2021) and mainly reverses m6A methylation (Wang et al., 2020b; Cai et al., 2021). ALKBH5 is an Fe^2+^ and Q-ketoglutarate-dependent non-heme oxygenase, belongs to the ALKB family, and only displays demethylation activity for m6A modifications on single-stranded RNA/DNA. ALKBH5 exerts essential biological functions in several tumors and cancers. Jin et al. (2019) reported that METTL3 and ALKBH5 modulate ITGA6 expression in BC cells to alter cell adhesion, thereby indicating the carcinogenic effects of m6A-modified ITGA6 and its regulatory mechanisms on BC initiation and development. In addition, down-regulated ALKBH5 expression in BC tissue and cell lines is related to a poor prognosis in patients with BC. ALKBH5 knockdown promotes BC cell proliferation, migration, and invasion, and reduces ciplatin chemosensitivity (Yu et al., 2021). ALKBH5 inhibits cancer progression in an m6A-dependent manner via the glycolytic pathway as mediated by casein kinase 2, and promotes BC cell sensitivity to cisplatin.

### 4.3 M6A Methylation is a Putative Prognostic Biomarker for BC

The diagnostic value of m6A-related regulatory proteins in BC is summarized (Table 2). Chen et al. collected 62 fresh bladder transitional BC samples (BC group) and 20 normal bladder mucosa specimens (controls). When compared with controls, WTAP expression was significantly increased in the BC group (Chen and Wang 2018). These authors identified a significant difference in the risk of disease recurrence between patients with negative WTAP protein expression levels and those with positive expression. In addition, MTTL3 (Han, et al., 2019), ALKBH5 (Yu, et al., 2021), m6A (Gu, et al., 2019), IGF2BP3 (Huang et al., 2020c), and FTO (Tao, et al., 2021) levels are closely related to prognosis in BC patients. However, no research has yet analyzed the diagnostic potential of m6A-related regulatory protein expression levels in urine and plasma.

**TABLE 2:** The potential of m6A as a diagnostic and prognostic tool in bladder cancer.

m6A regulator	Source	Detection method	Biomarker potential	References
WTAP	tissues	qRT-PCR, WB and IHC	A biomarker for prognosis	Hou et al. (2021)
METTL3	tissues	IHC	A biomarker for prognosis	Chen et al. (2021b)
ALKBH5	tissues	IHC	A biomarker for prognosis	Chang et al. (2021)
m6A	tissues	IHC	A biomarker for prognosis	Tsuchiya et al. (2021)
IGF2BP3	tissues	IHC	A biomarker for prognosis	Dai et al. (2021)
FTO	tissues	IHC	A biomarker for prognosis	Nie et al. (2021)

bladder cancer, BC; WTAP, WT1 associated protein; METTL3, methyltransferase 3, N6-adenosine-methyltransferase complex catalytic subunit.

METTL14, methyltransferase 14, N6-adenosine-methyltransferase subunit; ALKBH5, alkB homolog 5, RNA demethylase.

IGF2BP3, insulin like growth factor 2 mRNA binding protein 3; FTO, FTO alpha-ketoglutarate dependent dioxygenase.

qRT-PCR, quantitative real-time PCR; WB, Western blot; IHC, Immunohistochemistry.

## 5 Perspectives and Conclusion

M6A methylation mechanism have greatly contributed to the field of epigenetics. Methyltransferases, demethylases, and reading proteins jointly regulate m6A levels in downstream genes, thereby promoting tumor initiation and progression (Xu et al., 2020b; Yan et al., 2021). M6A RNA methylation comprises the m6A methyltransferase, m6A demethylase, and m6A binding proteins which regulate mRNA precursor shear, mRNA stability, and translation. M6A RNA methylation is related to tumor cell growth, metastasis, and drug resistance. There is no doubt that m6A methylation has significant potential for the development of new human cancer therapies. Additionally, bioinformatics show that m6A participates in BC *via* multiple biological processes: m6A regulators contribute to malignant progression and impact on prognoses (Chen et al., 2019d), m6A contributes to tumor microenvironments (Zhu et al., 2021b), and m6A regulates lncRNA in BC carcinogenesis (Li et al., 2021f). Moreover, bioinformatics tools can be used to study associations between m6A and BC. RMVar (Luo et al., 2021) and RMdisease (Chen et al., 2021c) presented the m6A-associated mutations in BC, and the BC-associated m6A sites were estimated by the heterogeneous network in DURM (Tang et al., 2019). However, challenges remain. Mechanisms underpinning m6A modulators in certain cancers are unclear, especially as so few studies on m6A modified “readers” exist. The evidence suggests that m6A modulators and related pathways could function as therapeutic targets, therefore, more input from the clinic is required to verify these therapeutic effects. Moreover, m6A modified proteins have the dual effect of suppressing or causing cancer, thus controversial research results must be fully explored to characterize these discrepancies. However, in the era of next-generation sequencing, the generation and analysis of big data (omics) will expand and transform cancer biology.

In summary, thanks to high-throughput sequencing and other biotechnologies, a clear role of m6A methylation during BC has emerged. METTL3, METTL14, ALKBH5, FTO, YTHDF1/3, YTHDF2, IGF2BP1, and IGF2BP3 aberrant expression occur in BC, mainly affect mRNA stability, and regulate the growth and metastasis of tumor cells. However, many challenges remain. The role of epigenetic networks in BC initiation and progression requires further exploration. It is vital to fully evaluate the safety and effectiveness of m6A-related regulatory factors and pathways as novel tumor therapy targets. Furthermore, exploring correlations between m6A and BC drug sensitivity and long-term prognostics is also essential.

## Abbreviations

m6A, N6-methyladenosine; mRNA, messenger RNA; 3′UTR, 3′untranslated region; snRNA, small nuclear RNA; APC, adenomatous polyposis coli; METTL3, methyltransferase 3, N6-adenosine-methyltransferase complex catalytic subunit; METTL14, methyltransferase 14, N6-adenosine-methyltransferase subunit; WTAP, WT1 associated protein; VIRMA, vir like m6A methyltransferase associated; RBM15, RNA binding motif protein 15; ZC3H13, zinc finger CCCH-type containing 13; CBLL1, Cbl proto-oncogene like 1; FTO, FTO alpha-ketoglutarate dependent dioxygenase; ALKBH5, alkB homolog 5, RNA demethylase; YTHDC1/2, YTH domain containing 1/2; YTHDF1/2/3, YTH N6-methyladenosine RNA binding protein 1/2/3; IGF2BP1/2/3, insulin like growth factor 2 mRNA binding protein 1/2/3.
