# Supplementary material for: Current Advances in N6-Methyladenosine Methylation Modification During Bladder Cancer
Source: Front Genet. 2022 Jan 11;12:825109. doi: 10.3389/fgene.2021.825109 (PMC8787278; doi:10.3389/fgene.2021.825109)
Supplement: Supplementary file 1 [file Table1.DOCX]

| **Table 1. The role of RNA m6A modification in bladder cancer** | | | |  |  |
| --- | --- | --- | --- | --- | --- |
|  |  |  |  |  |  |
| Type | m6A Regulator | Role in cancer | Biological function | Mechanism | Reference |
|  |  |  |  |  |  |
| m6A writer | METTL3 | Oncogene | Promotes cell growth and invasion | METTL3/AFF4/NF-κB/MYC | (Gao et al. 2020, Lan, Lu et al.) |
|  |  |  |  |  |  |
|  | METTL3 | Oncogene | Promotes malignant transformation | METTL3-m6A-CDCP1 | (Purslow, Nguyen et al.) |
|  |  |  | and tumorigenesis |  |  |
|  |  |  |  |  |  |
|  | METTL3 | Oncogene | Promotes cell proliferation | METTL3-DGCR8-PTEN | (Chen, Li et al.) |
|  |  |  |  | METTL3/pri-miR221/222 |  |
|  |  |  |  |  |  |
|  | METTL3 | Oncogene | Promotes cancer proliferation and metastasis | METTL3/YTHDF2/SETD7/KLF4 | (Wu, Yan et al.) |
|  |  |  |  |  |  |
|  | METTL3 | Oncogene | Promotes bladder cancer development | METTL3-m6A-CDCP1 | (Wollen, Hagen et al.) |
|  |  |  |  |  |  |
|  | METTL3 | Oncogene | Promotes oncogenesis and tumor angiogenesis | METTL3/TEK/VEGF-A | (Esteve-Puig, Climent et al.) |
|  |  |  |  |  |  |
|  | METTL3 | Oncogene | Promotes tumor proliferation and metastasis | cisplatin/METTL3/G-CSF | (Niu, Lin et al.) |
|  |  |  |  |  |  |
|  | METTL14 | Tumor suppressor | Inhibits the proliferation, self-renewal, metastasis | METTL14/m6A/NOTCH1 | (Cui, Zhang et al.) |
|  |  |  | and tumor initiating capacity of bladder TICs |  |  |
|  |  |  |  |  |  |
|  | METTL14 | Tumor suppressor | Inhibits cell invasion | ISO/FOXO3a/METTL14/Vimentin | (Nie, Zhang et al.) |
|  |  |  |  |  |  |
| m6A eraser | FTO | Tumor suppressor | Inhibits cell proliferation and invasion | — | (Li, Zhou et al.) |
|  |  |  |  |  |  |
|  | FTO | Oncogene | Promotes cancer initiation and progression | UPS18/FTO/PYCR1 | (Dai, Shi et al.) |
|  |  |  |  |  |  |
|  | FTO | Oncogene | Stimulates cell viability and tumorigenicity | FTO/MALAT/miR-384/MAL2 | (Tsuchiya, Yoshimura et al.) |
|  |  |  |  |  |  |
|  | ALKBH5 | Tumor suppressor | Inhibits bladder cancer growth and progression | ALKBH5/ITGA6/YTHDF1/3 | (Terajima, Lu et al.) |
|  |  |  |  |  |  |
|  | ALKBH5 | Tumor suppressor | Inhibits cell proliferation, migration, invasion, | ALKBH5/m6A/CK2a | (Xu, Wang et al.) |
|  |  |  | and increases cisplatin chemosensitivity |  |  |
|  |  |  |  |  |  |
| m6A reader | YTHDF1/3 | Oncogene | Promotes bladder cancer growth and progression | METTL3/ITGA6/YTHDF1/3 | (Hu, Pan et al.) |
|  |  |  |  |  |  |
|  | YTHDF2 | Oncogene | Promotes cancer proliferation and metastasis | METTL3/YTHDF2/SETD7/KLF4 | (Chang, Shi et al.) |
|  |  |  |  |  |  |
|  | IGF2BP1 | Oncogene | Promotes bladder cancer cell invasion, metastasis, | circPTPRA/ IGF2BP1/ FSCN1- MYC | (Hou, Zhang et al.) |
|  |  |  | and cell cycle progression |  |  |
|  |  |  |  |  |  |
|  | IGF2BP3 | Oncogene | Promotes cell proliferation, cell cycle, | IGF2BP3/JAK/STAT | (Kim and Siddiqui) |
|  |  |  | and inhibit apoptosis |  |  |
