# Supplementary material for: Current Advances in N6-Methyladenosine Methylation Modification During Bladder Cancer
Source: Front Genet. 2022 Jan 11;12:825109. doi: 10.3389/fgene.2021.825109 (PMC8787278; doi:10.3389/fgene.2021.825109)
Supplement: Supplementary file 2 [file Table2.DOCX]

| **Table 2. The potential of m6A as a diagnostic and prognostic tool in bladder cancer** | | |  |  |
| --- | --- | --- | --- | --- |
|  |  |  |  |  |
| m6A regulator | Source | Detection method | Biomarker potential | References |
|  |  |  |  |  |
| WTAP | tissues | qRT-PCR, WB and IHC | A biomarker for prognosis | (Hou, Zhang et al.) |
|  |  |  |  |  |
| METTL3 | tissues | IHC | A biomarker for prognosis | (Chen, Li et al.) |
|  |  |  |  |  |
| ALKBH5 | tissues | IHC | A biomarker for prognosis | (Chang, Shi et al.) |
|  |  |  |  |  |
| m6A | tissues | IHC | A biomarker for prognosis | (Tsuchiya, Yoshimura et al.) |
|  |  |  |  |  |
| IGF2BP3 | tissues | IHC | A biomarker for prognosis | (Dai, Shi et al.) |
|  |  |  |  |  |
| FTO | tissues | IHC | A biomarker for prognosis | (Nie, Zhang et al.) |
|  |  |  |  |  |
| bladder cancer, BC; WTAP, WT1 associated protein; METTL3, methyltransferase 3, N6-adenosine-methyltransferase complex catalytic subunit; | | | | |
| METTL14, methyltransferase 14, N6-adenosine-methyltransferase subunit; ALKBH5, alkB homolog 5, RNA demethylase; | | | | |
| IGF2BP3, insulin like growth factor 2 mRNA binding protein 3; FTO, FTO alpha-ketoglutarate dependent dioxygenase; | | | |  |
| qRT-PCR, quantitative real-time PCR; WB, Western blot; IHC, Immunohistochemistry. | | | |  |
